# Supplementary material for: Mechanism of ADP-Inhibited ATP Hydrolysis in Single Proton-Pumping FoF1-ATP Synthase Trapped in Solution
Source: Int J Mol Sci. 2023 May 8;24(9):8442. doi: 10.3390/ijms24098442 (PMC10178918; doi:10.3390/ijms24098442)
Supplement: Supplementary file 1 [file ijms-24-08442-s001.zip › ijms-2352652-supplementary.pdf]

Supporting Information

Mechanism of ADP-Inhibited ATP Hydrolysis in Single Proton-Pumping F_oF_1-ATP Synthase Trapped in Solution

Iván Pérez, Thomas Heitkamp and Michael Börsch

Single-Molecule Microscopy Group, Jena University Hospital, 07743 Jena, Germany

This supporting information contains 8 pages including one Table S1 and 7 Figures S1–S7.
Figures are numbered as referred to in the manuscript.

Table S1. Summary of active and inactive F_oF_1-ATP synthases at different ADP/ATP ratios

**[ATP]:[ADP]**	**Active**		**Inhibited**		**# fluctuations**	**ATP hydrolysis rate (ATP/s)**	
**(μM: μM)**	**# enzymes**	**%**	**# enzymes**	**%**	**(in active enzymes)**	**mean turnover per enzyme**	**calculated from rotation lengths**
100:0	49	21	180	79	386	92.1 ± 6	118.6 ± 3
90:10	38	18	169	82	292	90.7 ± 7	111.1 ± 6
80:20	28	25	84	75	202	96.9 ± 13	99.3 ± 6
70:30	27	19	112	81	234	87.9 ± 10	139.5 ± 6
60:40	31	20	126	80	282	89.1 ± 7	113.2 ± 6
50:50	15	9	148	91	88	83.6 ± 8	117.6 ± 9
40:60	12	12	91	88	110	73.3 ± 7	107.5 ± 9
30:70	14	8	155	92	55	86.5 ± 8	106.4 ± 15
20:80	2	2	113	98	6	-	-
10:90	3	2	146	98	13	-	-
0:100	2	2	115	98	8	-	-

**[ATP]**	**Active**		**Inhibited**		**# fluctuations**	**ATP hydrolysis rate (ATP/s)**	
**(μM)**	**# enzymes**	**%**	**# enzymes**	**%**		**Mean per enzyme**	**Rotations length**
5	60	26	170	74	415	68.9 ± 4	102.4 ± 2
20	84	33	170	67	657	66.5 ± 4	94.3 ± 3
40	106	31	236	69	908	113.5 ± 6	163.9 ± 1
100	94	36	165	64	822	128.8 ± 5	163.1 ± 1
1000	48	19	205	81	797	107.3 ± 7	107.5 ± 3

Figure S1. FRET time taces of single F_oF_1-ATP synthases at different ADP/ATP ratios (*part I*)

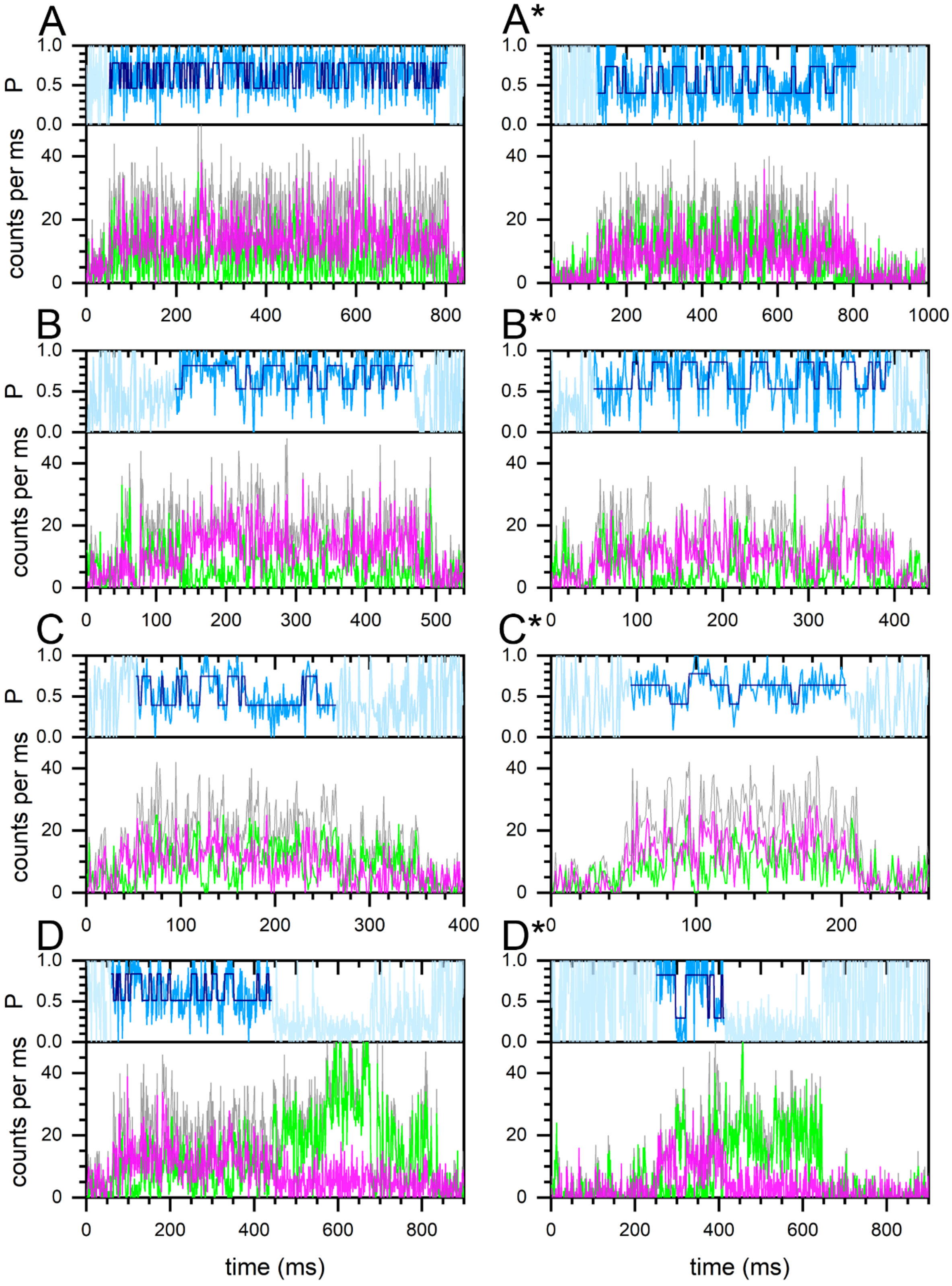

...

Figure S1. (*part II*)

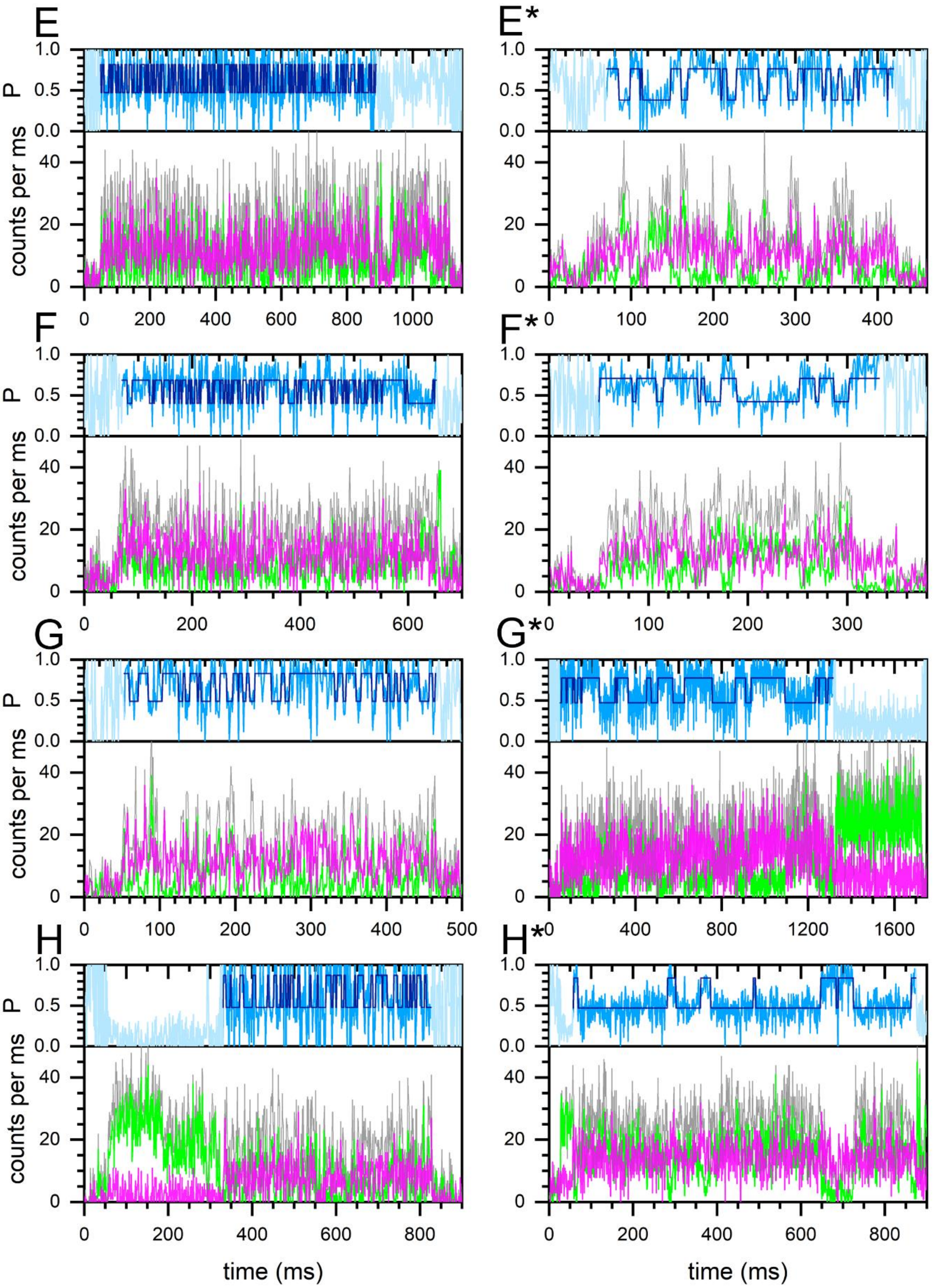

...

Figure S1. (*part III*)

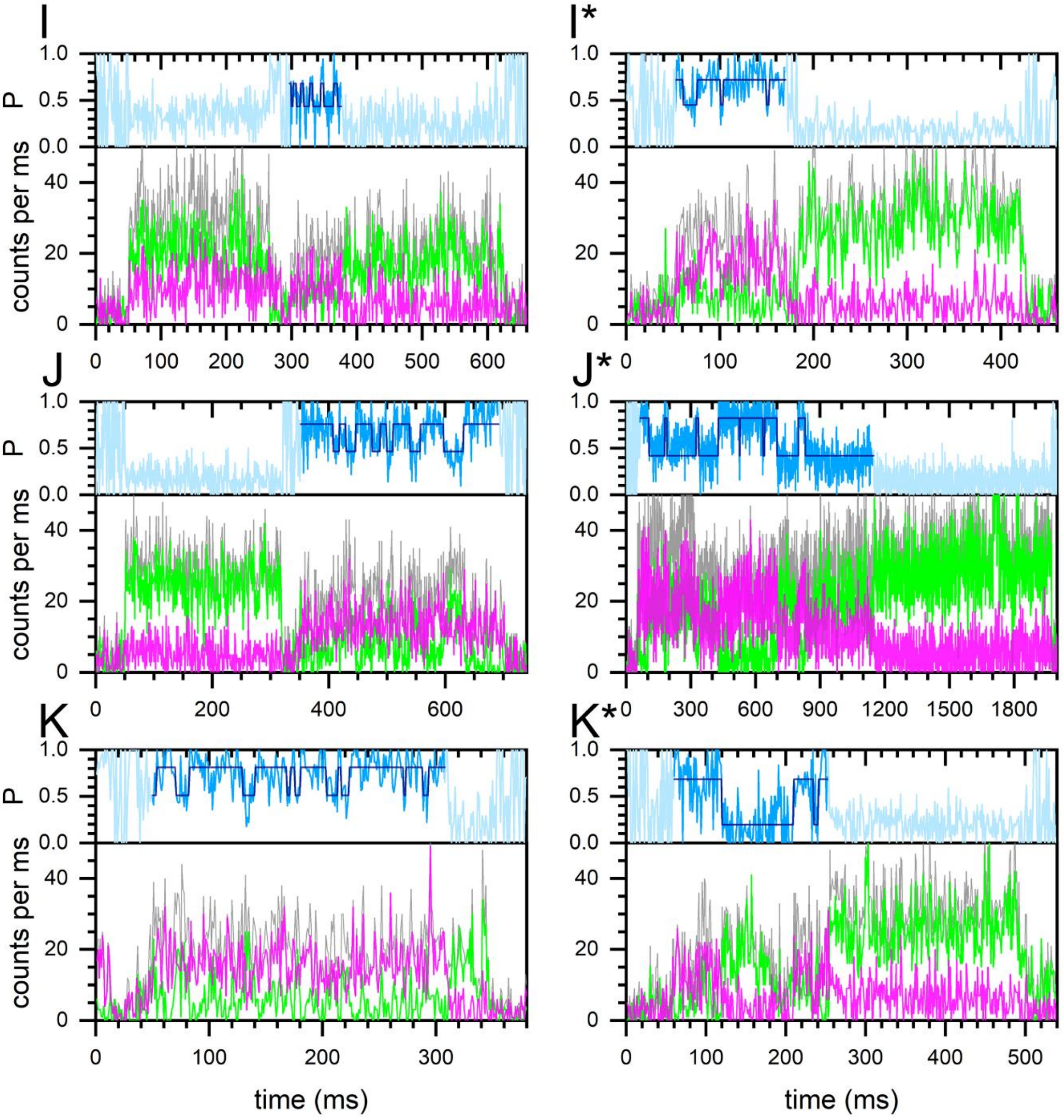

Figure S1. Turnover of single FRET-labeled F_oF_1-ATP synthases in the presence of 11 ADP/ATP ratios. The figure is split into three parts I to III. **Left** and **right** panels: time traces of F_oF_1-ATP synthases with either (**left**) fast or (**right**, marked with *) slow ε-subunit rotation. Recordings in the presence of (**A, A***) 0/100 ADP/ATP, (**B, B***) 10/90, (**C, C***) 20/80, (**D, D***) 30/70, (**E, E***) 40/60, (**F, F***) 50/50, (**G, G***) 60/40, (**H, H***) 70/30, (**I, I***) 80/20, (**J, J***) 90/10, and (**K, K***) 100/0 ADP/ATP; nucleotide concentrations in µM. FRET donor Cy3B photon counts per ms (green traces), FRET acceptor Alexa Fluor 647 photon counts (magenta traces), associated proximity factor P time trace in light blue, HMM-assigned FRET states in dark blue (HMM with two states). The sum intensities of FRET donor and acceptor photons are shown as gray traces.

The catalytic rates for each enzyme are given in the table below. Note that smFRET time traces D (fast) and D* (slow) are identical to Figures 2 A and B in the manuscript, and time traces H (fast) and H* (slow) are identical to Figures 3 A and B; the two corresponding rows are highlighted in the table.

smFRET time trace in Figure S1	[ATP]:[ADP]	fluctuating FRET states period in ms (rotating ε-subunit)		# of rotations in FRET bursts		mean ATP hydrolysis rate for the active enzymes (ATP/s)	
		fast	slow (*)	fast	slow (*)	fast	slow (*)
A, A*	100:0	839	272	58	8	209.1	88.2
B, B*	90:10	557	214	29	6	156.2	84.1
C, C*	80:20	385	1235	19	13	148.1	31.6
D, D*	70:30	465	581	26	5	167.7	25.8
E, E*	60:40	727	605	38	13	158.8	64.5
F, F*	50:50	295	309	9	9	91.5	87.4
G, G*	40:60	172	76	5	2	87.2	78.9
H, H*	30:70	358	154	13	3	108.9	58.4
I, I*	20:80	62	77	4	2	193.5	77.9
K, K*	10:90	213	621	5	4	70.4	19.3
L, L*	0:100	240	31	8	1	99.9	96.7

Figure S2. Periods of fluctuating FRET states at different ADP/ATP ratios

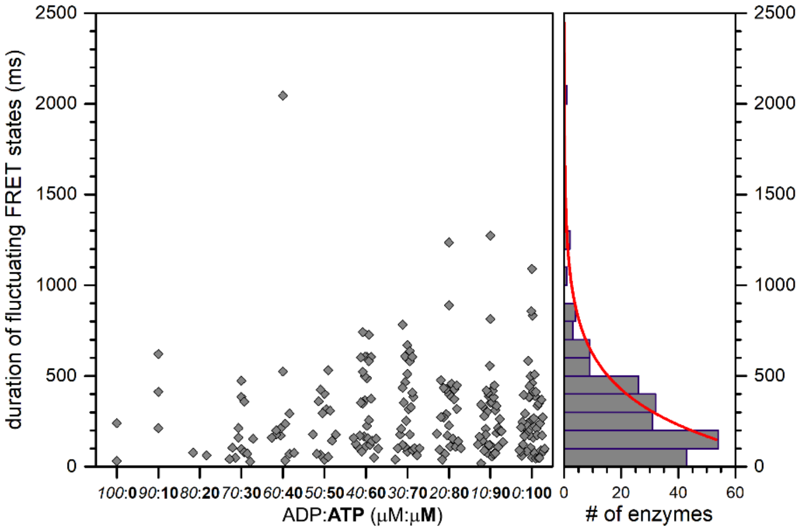

Figure S2. Scatter plot of the duration of the individual fluctuating FRET state periods within the photon bursts of FRET-labeled F_oF_1-ATP synthases in the presence of 11 different ADP/ATP ratios. Fluctuating FRET states indicated the active, ATP-hydrolyzing enzymes (black diamonds). Right, histogram of the durations of the individual fluctuating FRET state periods using 100 ms binning. The monoexponential decay fit (red curve) yields a 1/e time of 283±22 ms.

Figure S3. Periods of fluctuating FRET states at different ATP concentrations

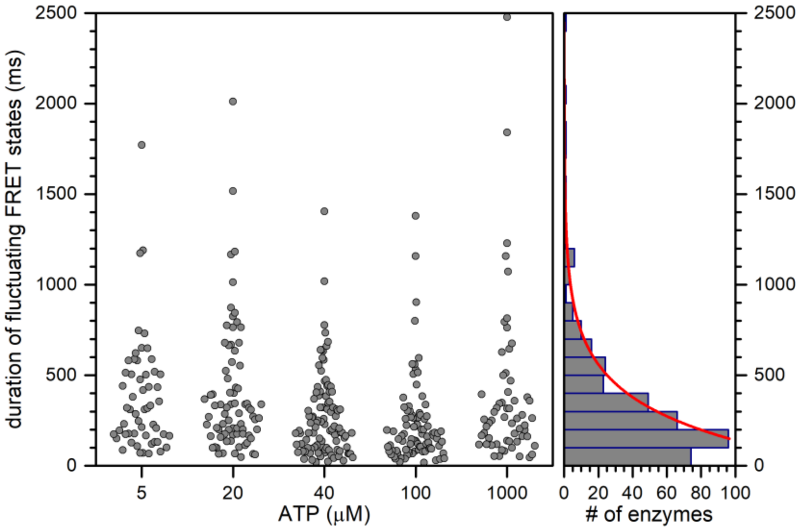

Figure S3. Scatter plot of the duration of the individual fluctuating FRET state periods within the photon bursts of FRET-labeled F_oF_1-ATP synthases in the presence of 5 different ATP concentrations. Note that these are re-analyzed smFRET data from our previous measurements [1]. Fluctuating FRET states in the smFRET data indicated the active, ATP-hydrolyzing enzymes (black circles). Right, histogram of the durations of the individual fluctuating FRET state periods using 100 ms binning. The monoexponential decay fit (red curve) yields a 1/e time of 261±10 ms.

Figure S4. Durations of non-fluctuating FRET states at different ADP/ATP ratios

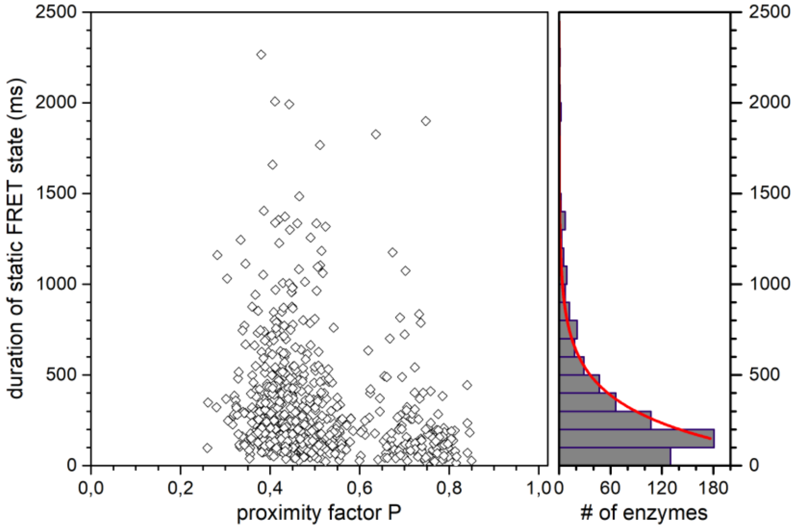

Figure S4. 2D scatter plot of the duration of the non-fluctuating static FRET states with different proximity factor P of FRET-labeled F_oF_1-ATP synthases, combined for all 11 different ADP/ATP concentration ratios. Right, histogram of the durations of the non-fluctuating static FRET states using 100 ms binning. The monoexponential decay fit (red curve) yields a 1/e time of 222±7 ms.

Figure S5. Durations of non-fluctuating FRET states at different ATP concentrations

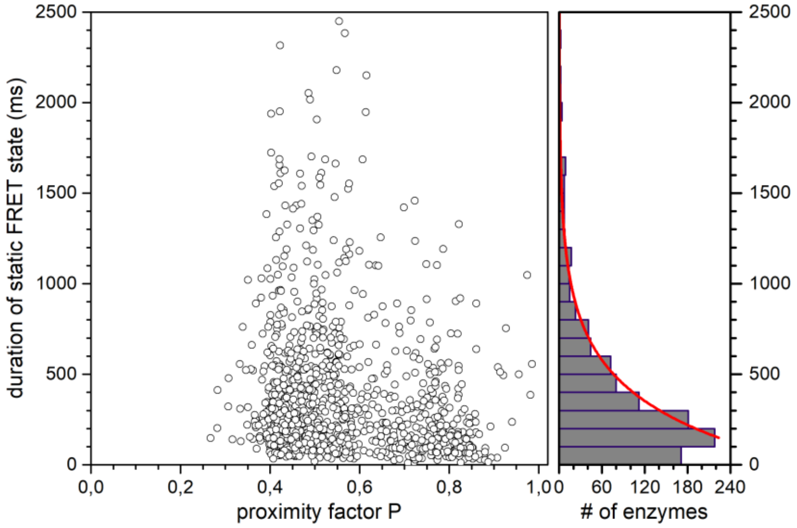

Figure S5. 2D scatter plot of the duration of the non-fluctuating static FRET states with different proximity factor P of FRET-labeled FoF1-ATP synthases, combined for all 5 different ATP concentrations ("ATP only"). Note that these are re-analyzed smFRET data from our previous measurements [1]. Right, histogram of the duration of the non-fluctuating static FRET states using 100 ms binning. The monoexponential decay fit (red curve) yields a 1/e time of 327±10 ms.

Figure S6. Individual ATP hydrolysis rates at different ATP concentrations

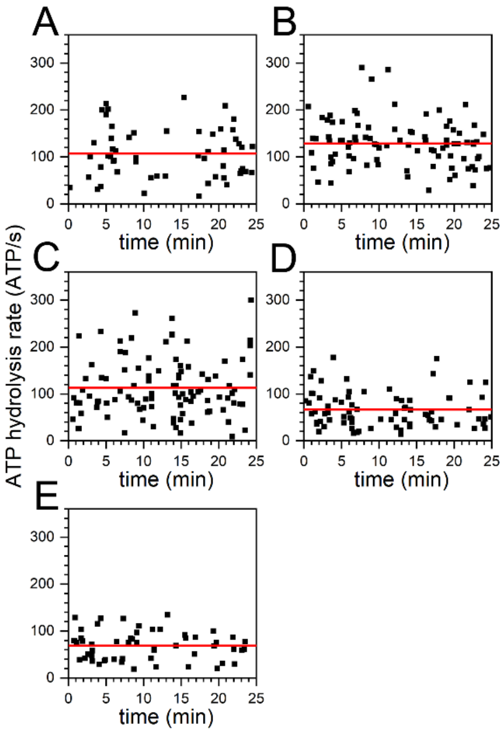

Figure S6. Mean turnover of individual FRET-labelled F_oF_1-ATP synthases (black squares) in the presence of 5 different ATP concentrations observed in the first 25 min recording time after addition of ATP. The ATP concentrations were (**A**) 1 mM, (**B**) 100 µM, (**C**) 40 µM, (**D**) 20 µM and (E) 5 µM. Note that these are re-analyzed smFRET data from our previous measurements [1]. Red lines indicate the mean turnover, i.e., for (**A**) 107 ATP/s, for (**B**) 129 ATP/s, for (**C**) 114 ATP/s, (**D**) 67 ATP/s and for (**E**) 69 ATP/s (see Table S1 above).

Figure S7. Durations of fluctuating FRET state pairs at different ATP concentrations

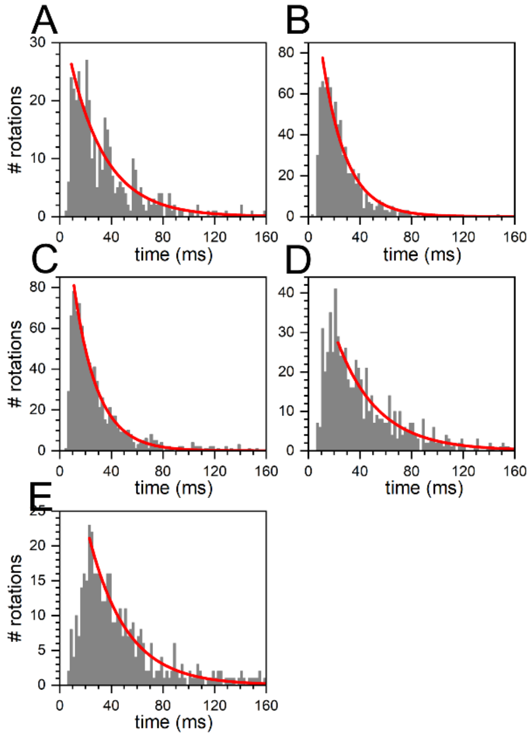

Figure S7. Durations of fluctuating FRET state pairs in smFRET time traces. Each FRET state pairs comprised one high FRET plus one sub-sequent low FRET state. Each FRET state pair represented a full rotation of the ε-subunit of the F_oF_1-ATP synthases in the presence of 5 different ATP concentrations. The ATP concentrations were (**A**) 1 mM, (**B**) 100 µM, (**C**) 40 µM, (**D**) 20 µM and (E) 5 µM. Note that these are re-analyzed smFRET data from our previous measurements [1]. The red curves were monexponential fits yielding average ATP hydrolysis rates of (**A**) 108 ATP/s, (**B**) 163 ATP/s, (**C**) 164 ATP/s, (**D**) 94 ATP/s and (**E**) 102 ATP/s (see Table S1 above).

Reference

[1] Heitkamp, T.; Börsch, M., Fast ATP-Dependent Subunit Rotation in Reconstituted F_oF_1-ATP Synthase Trapped in Solution. *J Phys Chem B* **2021**, 125, 7638-7650.
